# A phase I study of the combination of atezolizumab, tiragolumab, and stereotactic body radiation therapy in patients with metastatic multiorgan cancer

**DOI:** 10.1186/s12885-023-11534-6

**Published:** 2023-11-09

**Authors:** Nicolas Roussot, Jean-David Fumet, Emeric Limagne, Marion Thibaudin, Alice Hervieu, Audrey Hennequin, Sylvie Zanetta, Lorraine Dalens, Théo Fourrier, Loick Galland, Pierre Jacob, Aurélie Bertaut, Emilie Rederstorff, Cédric Chevalier, Sarah Ghirardi, Elodie Gilbert, Azzat Khoukaz, Etienne Martin, Constance Nicolet, Magali Quivrin, David Thibouw, Noémie Vulquin, Gilles Truc, Magali Rouffiac, Francois Ghiringhelli, Céline Mirjolet

**Affiliations:** 1 Department of Medical Oncology, Center Georges François Leclerc, 1 rue du Professeur Marion, Dijon, 21000 France; 2 Department of Radiotherapy, Center GF Leclerc, Dijon, France; 3 Cancer Biology Transfer Platform, Dijon, France; 4 GIMI Genetic and Immunology Medical Institute, Dijon, France; 5 University of Burgundy-Franche Comté, Dijon, France; 6 UMR INSERM 1231, Dijon, France; 7 Department of Epidemiology and Biostatistics, Center GF Leclerc, Dijon, France; 8 Radiation Oncology Department, Preclinical Radiation Therapy and Radiobiology Unit, Center GF Leclerc, Unicancer, Dijon, France

**Keywords:** Non-small cell lung cancer, Renal cell cancer, Bladder cancer, Head and neck cancer, PDL-1, TIGIT, Atezolizumab, Tiragolumab, Stereotactic body radiation therapy, SBRT, Immunotherapy

## Abstract

**Background:**

Immunotherapy targeting the PD-1/PD-L1 pathway is a standard of care in a number of metastatic malignancies, but less than a fifth of patients are expected to respond to ICIs (Immune Checkpoint Inhibitors). In a clinical trial, combining the anti-TIGIT (*T cell immunoreceptor with Ig and ITIM domains*) Mab (monoclonal antibody) tiragolumab with atezolizumab improved outcomes in non-small cell lung cancer. In preclinical models, SBRT (Stereotactic Body Radiation Therapy) could increase expression levels of the inhibitory co-receptors TIGIT and PD-L1. We aim to assess the combination of tiragolumab with atezolizumab and SBRT in metastatic, previously treated by ICIs, non-small cell lung cancer, head and neck cancer, bladder cancer, and renal cell cancer.

**Methods:**

This phase I study (ClinicalTrials.gov NCT05259319) will assess the efficacy and safety of the combination of atezolizumab with tiragolumab and stereotactic body radiation therapy in patients with histologically proven metastatic non-small cell lung cancer, renal cell cancer, bladder cancer, and head and neck cancer previously treated. First part: 2 different schedules of SBRT in association with a fixed dose of atezolizumab and tiragolumab will be investigated only with metastatic non-small cell lung cancer patients (cohort 1). The expansion cohorts phase will be a multicentric, open-label study at the recommended scheme of administration and enroll additional patients with metastatic bladder cancer, renal cell cancer, and head and neck cancer (cohort 2, 3 and 4). Patients will be treated until disease progression, unacceptable toxicity, intercurrent conditions that preclude continuation of treatment, or patient refusal in the absence of progression or intolerance. The primary endpoint of the first phase is the safety of the combination in a sequential or concomitant scheme and to determine the expansion cohorts phase recommended scheme of administration. The primary endpoint of phase II is to evaluate the efficacy of tiragolumab + atezolizumab + SBRT in terms of 6-month PFS (Progression-Free Survival). Ancillary analyses will be performed with peripheral and intratumoral immune biomarker assessments.

**Trial registration:**

This study is registered on ClinicalTrials.gov: NCT05259319, since February 28th, 2022.

**Supplementary Information:**

The online version contains supplementary material available at 10.1186/s12885-023-11534-6.

## Background

ICIs are a key compound in the therapeutic arsenal approved in the metastatic setting for a number of solid malignancies [1–5]. Mabs targeting the immunological synapse currently constitute the first-line standard of care treatment for metastatic non-small lung and head and neck cancer in monotherapy or in combination with chemotherapy [6–8]. Likewise, in the first line setting for metastatic renal clear cell carcinoma, several strategies are available with ICIs combination or ICIs and anti-angiogenic targeted therapies [3]. In the context of metastatic urothelial cancer, ICIs showed a clinical benefit either in maintenance after first-line chemotherapy with no disease progression or in the second-line setting [9, 10]. Although immunotherapy alone or in combination with chemotherapy has revolutionized the treatment of several malignancies, allowing a dramatic OS (Overall Survival) enhancement with a good safety profile, a large proportion of patients are still resistant to such therapies [11].

This lack of efficacy of ICIs has been intensively explored, looking for answers within the tumor microenvironment (TME) and the characterization of tumor-infiltrating lymphocytes (TILs), which brought out the ‘cold’ and ‘hot’ tumor concepts. Considering theses evidences, which testify the central role of the immune context, signatures have been designed and have demonstrated their prognostic values reaching a superior accuracy than the classical AJCC/UICC TNM [12]. Beyond the classical immune checkpoints PD-1, PD-L1, and CTLA4, additional immunomodulatory pathways have been characterized and are currently targeted in order to enhance immunotherapy efficacy. One of these co-inhibitory receptors is the receptor TIGIT which down-regulates T cell and NK function by interacting with its ligands CD155 and CD122 [13]. Tiragolumab is a Mab that binds TIGIT at the immune cell surface, thus preventing inhibitory signal transduction. In the first-line setting of metastatic non-small cell lung cancer, combining tiragolumab with atezolizumab demonstrated a better overall response rate (ORR) and longer PFS than anti-PD-L1 monotherapy, along with a tolerable safety profile [14, 15].

Radiotherapy (RT) has been shown to trigger immunogenic cell death with HMGB1 (High Mobility Group Box 1) release by tumor cells and dendritic cells (DCs) activation through TLR4, allowing a tumor antigen-specific T cell immunity [16]. This is one of the mechanisms that may support the abscopal effect induced by radiation therapy. The abscopal effect is based on the ability of radiation to generate neoantigens (TNAs) expression by tumor cells, allowing their capture by antigen- presenting cells (APCs) such as DCs, which could subsequently prime T cells in secondary lymphoid organs. After activation, migration, and infiltration in both irradiated and non-irradiated tumor metastases, T cells may exert an anti-tumor immune response. Accordingly, combining immunotherapy with radiotherapy is an interesting approach to enhance the abscopal effect [17]. Aware that metastatic lesions are heterogeneous within their different location sites, it is thought that multisite irradiation should be more potent than single-site irradiation in order to unveil more tumor-associated antigens (TAAs) and/or TNAs, thus enhancing the synergic effect between radiotherapy and ICI [18]. Reassuringly, such a combination of SBRT (Stereotactic Body Radiation Therapy) with immunotherapy in the metastatic setting appears to be safe with acceptable toxicity [19]. Although this association shows great promise, it requires optimization of factors such as administration schedule, fractionation, and immunotherapy types.

In preclinical models of CT26 and B16 murine cancers, we assessed the effect of several radiotherapy doses per fraction on PD-L1 and TIGIT expression levels. Results suggest that irradiation using a moderate hypofractionation schedule is more likely to trigger PD-L1 and TIGIT expression, justifying our therapeutic approach that targets both receptors. Furthermore, the triple association of anti-TIGIT, anti-PD-L1, and concomitant RT with a 3 fractions of 8 Gy schedule was correlated with better survival in this model compared to all other 3x8 Gy regimens, i.e., anti-PD-L1 plus RT 3x8 Gy, anti-TIGIT plus RT 3x8 Gy, and RT 3x8 Gy alone [20].

**Fig. 1:**
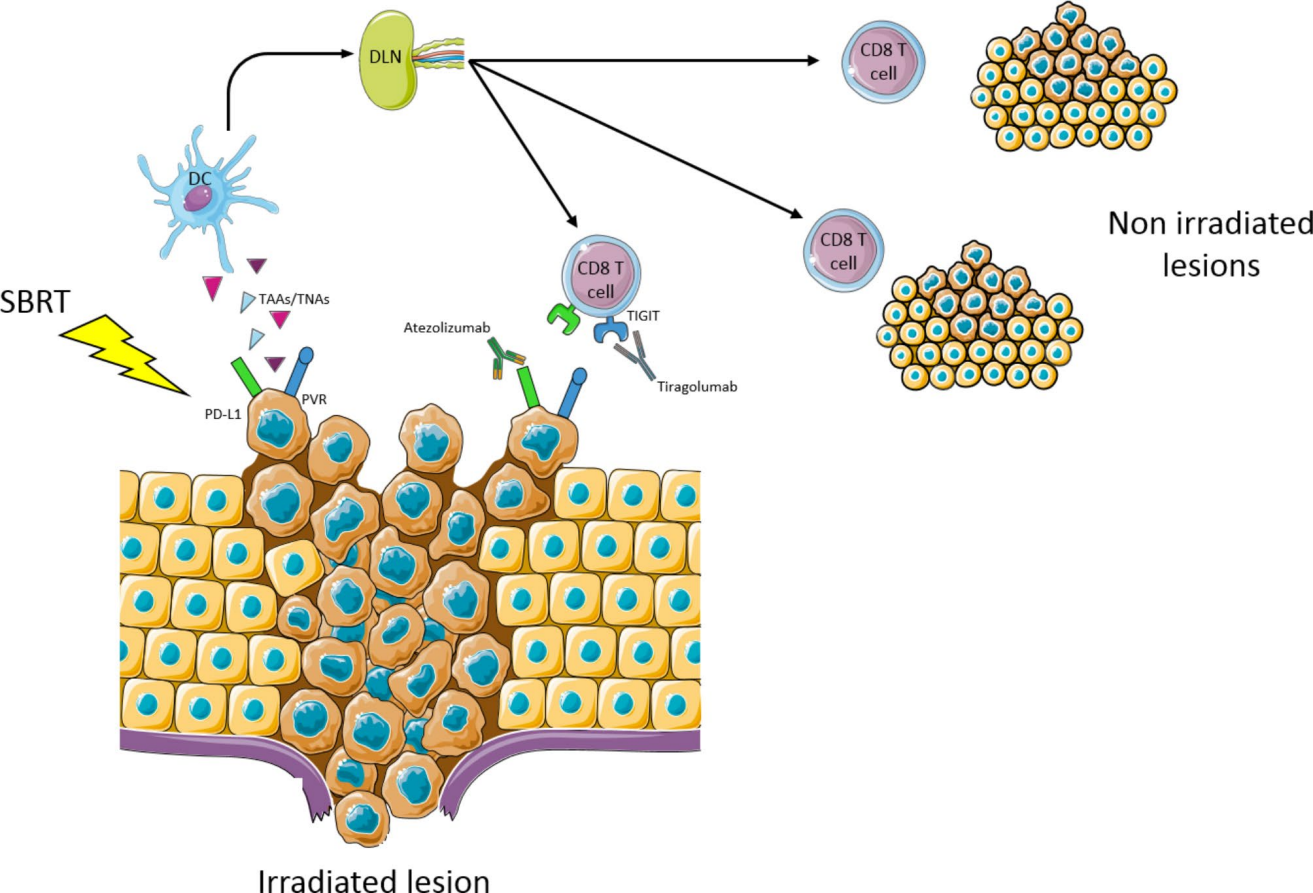
Rationale: SBRT induces immunogenic cell death, which promotes TAAs and/or TNAs uptake by DCs. Then DCs migrate into the DLN in order to prime naive T cells. After activation in the DLN, CD8 T cells join irradiated and non-irradiated lesions to perform their anti-tumor immune function. CD8 T cell: CD8 T lymphocyte; DC: dendritic cell; DLN: draining lymph node; PD-L1: programmed death-ligand 1; PVR: poliovirus receptor; SBRT: stereotactic body radiation therapy; TAA: tumor-associated antigen; TNA: tumor neoantigen; TIGIT: T cell immunoreceptor with Ig and ITIM domains

Thus, based on the clinical trial association of tiragolumab and atezolizumab and our preclinical data with additional SBRT, we believe that a trial combining these therapies will be important to confirm the safety and efficacy of such an approach. The rationale supporting this study is provided in Fig. 1.

## Methods

### Study design

This trial will include two parts:

The first part will be a monocentric, open-label, phase I, with the aim of establishing the recommended safety scheme of administration (concomitant or sequential) of tiragolumab + atezolizumab + SBRT. Only patients suffering from metastatic non-small cell lung cancer (cohort 1) will be enrolled in phase I. Other cancers will be studied in the second part of the study.

The second part will be a multicentric study, open-label, expansion cohorts phase under the recommended scheme of administration. Patients from 4 different cohorts (metastatic non-small cell lung cancer, metastatic bladder cancer, metastatic renal cell carcinoma, and metastatic head and neck carcinoma) will be enrolled in the second part.

Patients will be treated until disease progression, unacceptable toxicity, intercurrent conditions that preclude continuation of treatment, or patient refusal in the absence of progression or intolerance. The maximum duration of the treatment with immunotherapy will be 24 months.

### Phase I design

Two different schemes of administration will be investigated:

- Sequential administration (scheme 1): SBRT (3 fractions of 8 Gy in 5 days) starting on week 1, followed by the first injection of atezolizumab + tiragolumab at week 2, then every 21 days.
- Concomitant administration (schema 2): SBRT (3 fractions of 8 Gy in 5 days) starting on week 1, with the first injection of atezolizumab + tiragolumab concomitant to the first radiotherapy fraction, then every 21 days.

**Fig. 2:**
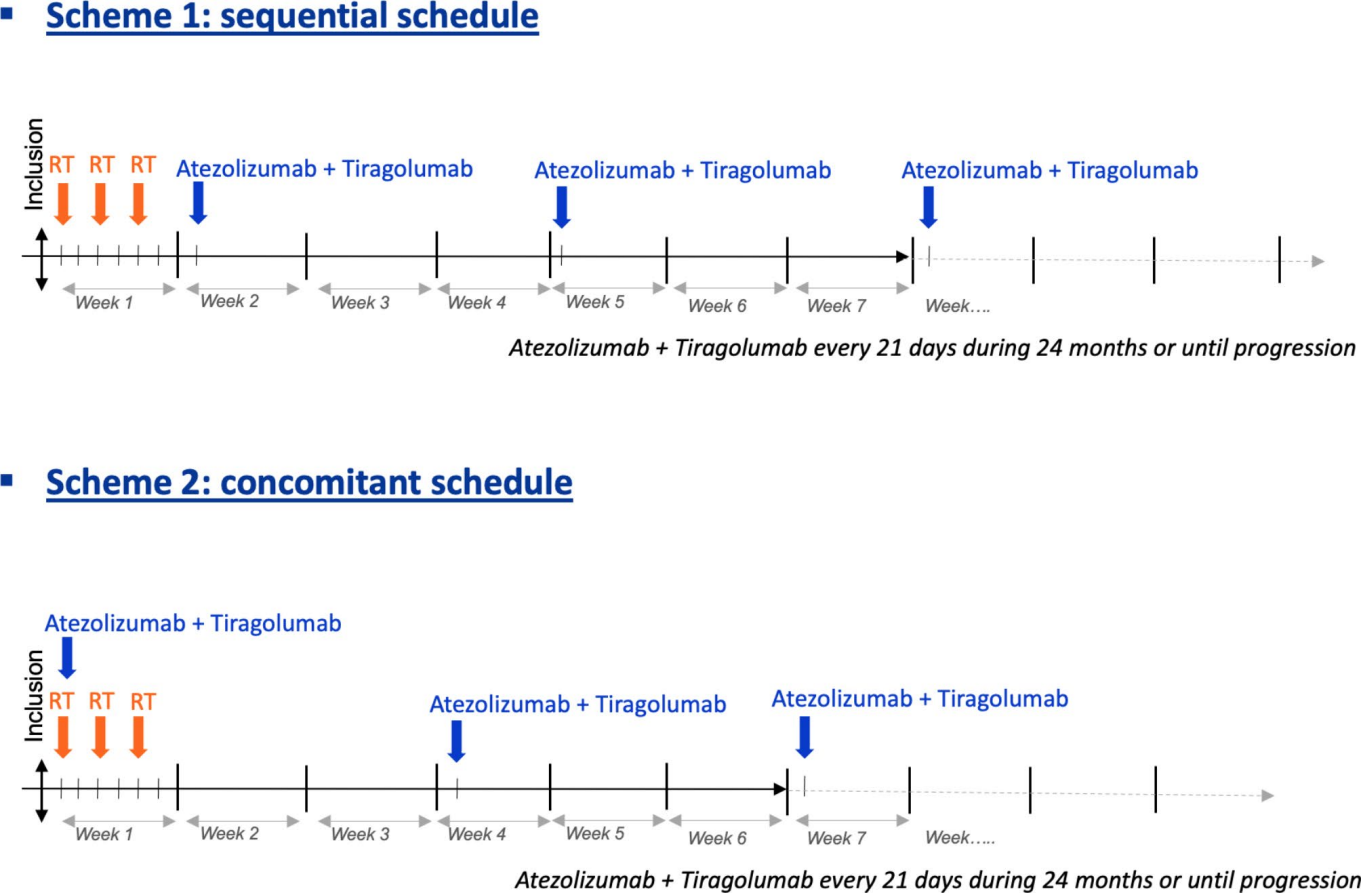
Study design. Sequential schedule (top panel), concomitant schedule (bottom panel)

Immunotherapies will be administered intravenously: atezolizumab (1200 mg) first, then tiragolumab (600 mg) second. The fixed dose of 600 mg was selected on the basis of available clinical pharmacokinetics, efficacy, and safety data from the combined phase Ia/phase I study (GO30103), with single-agent tiragolumab or tiragolumab in combination with atezolizumab. This dose regimen was used in the CITYSCAPE trial that assessed the atezolizumab and tiragolumab combination in metastatic NSCLC [14, 15]. The study design is provided in Fig. 2.

### Extension cohorts phase design

Sequential or concomitant administration based on the phase I results.

### Progression of the study

The study will start with a 28-day baseline phase.

The end of the study is defined as the date of the last follow-up visit performed by the last patient in the study.

The planned follow-up treatment phase will be 24 months after the last dose of study treatment.

The total duration of the study will be approximately 96 months, including about 48 months of active enrollment (24 months for phase I and 24 months for the expansion cohorts phase), 24 months of treatment, and 24 months of follow-up.

### Expected treatment-related adverse events

The possible toxicity of a combination of radiotherapy and immunotherapy mainly depends on the location of the irradiation and may involve hepatitis for liver irradiation or autoimmune pneumonitis for lung irradiation. However, such side effects are transitory and manageable. In a phase I study, administration of SBRT before immunotherapy appeared safe with acceptable toxicity. The most common treatment-related adverse events were general disorders and administration site abnormalities. Other frequent mild adverse events (> 20%) included gastrointestinal disorders, metabolism and nutrition disorders, musculoskeletal and connective tissue disorders, and respiratory, thoracic, and mediastinal disorders [19].

### Dose modifications

There will be no dose modifications, including dose reductions for tiragolumab, atezolizumab, or radiotherapy, in this study.

### Management of Adverse Events and treatment interruption

Guidelines for the management of AEs are provided in the supplementary appendix [see Additional file 1]. Atezolizumab and/or tiragolumab may be temporarily suspended as appropriate for the management of toxicity. On the basis of the available characterization of the mechanism of action, tiragolumab may cause adverse events similar to but independent of atezolizumab, may exacerbate the frequency or severity of atezolizumab-related adverse events, or may have non-overlapping toxicities with atezolizumab. Because these scenarios may not be distinguished from one another in the clinical setting, immune-mediated adverse events should generally be attributed to both study drugs, and dose interruptions or treatment discontinuations in response to immune-mediated adverse events should be applied to both tiragolumab and atezolizumab.

Tiragolumab and atezolizumab may be held for a maximum of 12 weeks (or approximately four cycles).

If tiragolumab is interrupted for more than approximately 12 weeks for any reason, the patient will have to permanently discontinue tiragolumab treatment but may continue atezolizumab if there is no contraindication and after discussion with the coordinating investigator to determine whether the toxicity is considered related to tiragolumab and/or to the combination. Continued dosing with single-agent atezolizumab administered to patients will require that all other study eligibility criteria continue to be met. An exception can be made if, in the judgment of the investigator, the patient is likely to derive clinical benefit from resuming tiragolumab after a hold longer than 12 weeks. In this case, tiragolumab may be restarted with the approval of the coordinator of the study by contacting the sponsor for advice.

If atezolizumab is interrupted for more than approximately 12 weeks (or approximately four cycles), the patient will have to permanently discontinue atezolizumab. However, if, in the judgment of the investigator, the patient is likely to derive clinical benefit from atezolizumab after a hold longer than 12 weeks, atezolizumab may be restarted with the approval of the coordinator of the study by contacting the sponsor for advice.

If a patient must be tapered off steroids used to treat adverse events, atezolizumab may be withheld for an additional time beyond approximately 12 weeks from the last dose, and tiragolumab may be withheld for an additional time beyond approximately 12 weeks from the last dose until steroids are discontinued or until steroids are reduced to a prednisone dose (or dose equivalent) ≤ 10 mg/day. The acceptable length of interruption will depend on an agreement between the investigator and the coordinator of the study after contacting the sponsor for advice and approval. Dose interruptions for reason(s) other than toxicity, such as surgical procedures, may be allowed with the coordinator of the study by contacting the sponsor for advice and approval.

After both tiragolumab and atezolizumab have been permanently discontinued, the patient will be monitored for safety and efficacy.

### Study objectives

#### Main objective of the phase I part

The main objective of the phase I study is to evaluate the safety of the SBRT, atezolizumab and tiragolumab Mabs combination in a sequential or concomitant scheme and determine the recommended expansion cohorts phase scheme of administration.

#### Main objective of the expansion cohorts phase

The main objective of the study is to evaluate, among patients treated with the recommended scheme, the efficacy of the SBRT, atezolizumab, and tiragolumab combination in terms of 6-month progression-free survival (PFS).

#### Secondary objective of the expansion cohorts phase

The secondary objectives will be determined by the recommended scheme of administration.

1. To evaluate the progression-free survival (PFS) following SBRT, atezolizumab, and tiragolumab combination
2. To evaluate the long-term safety of SBRT, atezolizumab, and tiragolumab antibodies combination
3. To evaluate the overall survival (OS) following SBRT, atezolizumab, and tiragolumab combination
4. To evaluate the overall response rate (ORR) and non-progression rate (NPR) following SBRT, atezolizumab and tiragolumab combination
5. To evaluate the duration of response (DOR) and non-progression duration (NPD) following SBRT, atezolizumab and tiragolumab combination
6. To evaluate the abscopal effect (non-irradiated lesions volume variation).

### Exploratory objectives

1. To evaluate efficacy as a function of PD-L1, TIGIT, CD155, and CD112 expression
2. To evaluate the evolution of the in situ immune response
3. To evaluate the evolution of the systemic immune response
4. To evaluate the induction of neoantigen and tumor-shared antigen-specific immune response
5. To evaluate the radiotherapy planning target volumes (PTV) impact on systemic immune response
6. To create a research biosample repository (blood and tumor)

### Study endpoints

#### Primary end-points of phase I

Safety will be evaluated using DLTs (Dose-Limiting Toxicities) that occur:

- the first 5 weeks (35 days) after the first dose of study treatment for **sequential** administration (3x8 Gy of radiotherapy and 3 dosings of immunotherapy)
- the first 4 weeks (28 days) after the first dose of study treatment for **concomitant** administration (3x8 Gy of radiotherapy and 3 dosings of immunotherapy).

**Fig. 3:**
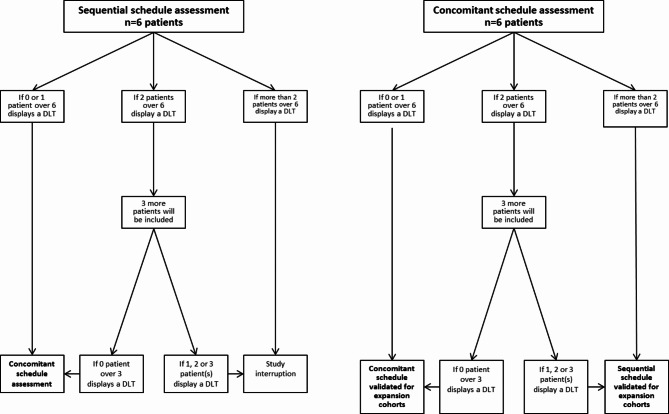
Decision diagram. Algorithm for assessment of sequential schedule (left panel) and concomitant schedule (right panel)

DLTs are defined by all events that are deemed related to the study treatments (SBRT and/or tiragolumab and/or atezolizumab), unless there is a clear alternative cause, with CTCAE version 5.0 grade ≥ 3 non-resolutive (return to basal grade) after 14 days. Any grade 5 related to study treatment will be considered a DLT. No clinical trial has combined SBRT, anti-PD-L1, and anti-TIGIT yet. In order to reduce toxicity risk, we will use a non-ablative SBRT dose, and we will begin this trial by first evaluating tolerance using two types of combination schedules. For the first one, immunotherapies will be administered one week after the start of SBRT with at least 2 days between the end of radiotherapy and the injection of immunotherapy. If no DLT is highlighted, we will evaluate the tolerance of our combination administered using the concomitant scheme. The concomitant scheme was designed on the basis of our preliminary data obtained with RT delivered on the same day as immunotherapy initiation. The decision diagram provided in Fig. 3 describes how the scheme of administration will be selected (sequential or concomitant). If safety is validated with the sequential schedule, the concomitant schedule will be assessed. If safety is then validated with the concomitant schedule, this scheme of administration will be selected for expansion cohorts. Otherwise, the sequential schedule will be selected for expansion cohorts. If there is no difference in toxicity, the concomitant schedule will be selected. Safety is defined according to the decision diagram.

For the **sequential schedule**, safety is defined by:

- 0 or 1 patient over 6 displays a DLT
- **or** 2 patients over 6 display a DLT, then 0 over 3 new included patients display a DLT

For the **concomitant schedule**, safety is defined by:

- 0 or 1 patient over 6 displays a DLT
- **or** 2 patients over 6 display a DLT, then 0 over 3 new included patients display a DLT

#### Primary endpoint of the expansion cohorts phase

Efficacy will be evaluated using the 6-month PFS. PFS is defined as the time from inclusion to the first occurrence of disease progression, as determined by the investigator according to RECIST v1.1 and iRECIST, or death from any cause, whichever occurs first. It will be evaluated at 6 months.

#### Secondary endpoints

Every secondary endpoint will be determined for patients included in the recommended scheme administration (expansion phase):

1. PFS according to RECIST v1.1 and iRECIST, or death from any cause, whichever occurs first. It will be evaluated at 12 months. Additionally, the median will also be displayed.
2. Long-term safety of the SBRT, atezolizumab, and tiragolumab antibody combination will be evaluated with acute and late adverse events (AAEs) according to CTCAE 5.0.
  Acute adverse events are adverse events occurring within the first sixth months, and late adverse events are adverse events occurring after the first six months.
3. Overall survival (OS) is defined as the time from inclusion to death from any cause.
4. Overall response rate (ORR) and non-progression rate (NPR) are evaluated from the first two evaluation scanner images carried out at 9 and 18 weeks after time from inclusion (CT-scan forecast before the 3rd and 6th cure) as determined by the investigator according to RECIST v1.1 and iRECIST. Overall response is defined by a partial or complete response. Non-progression is defined by a partial or complete response and a stable disease.
5. Duration of response (DOR) is defined as the time from documentation of a disease complete or partial response to disease progression, defined according to RECIST v1.1 and iRECIST. Non-progression duration (NPD) is defined as the time from documentation of a complete response, partial response, or stable disease to a disease progression, as defined according to RECIST v1.1 and iRECIST.
6. Abscopal effect is evaluated following non-irradiated metastasis volume(s) from scanner images.

### Exploratory endpoint

1. Immunohistology for PDL1 (Sp142 and 22C3), TIGIT, CD155, and CD112
2. Immunohistology before treatment and after 2 months of therapy to study immune infiltrates (PDL1, CD112, CD155, CD8, CD19, DC-Lamp, TCF1, CD163) using multiplex immune histology and spatial transcriptomic profiling for a sample of good responders or stable and poor responders (progressing straight away)
3. Study of peripheral immune response upon multiparametric cytometry and multiplex cytokine testing at baseline and following treatment
4. Study of a specific peripheral immune response against two shared antigens (telomerase and NY-ESO1) on isolated peripheral blood mononuclear cells (PMBC). Telomerase and NY-ESO1-specific T-cell responses will be assessed by a standardized IFN-γ ELISPOT assay.
5. Neopeptide detection upon exome sequencing
6. Study of the impact of the total volume receiving the prescribed dose on specific tumor immune response

### Study population and eligibility criteria

Patients will be included in different cohorts, depending on the type of histologically proven metastatic cancer:

- Cohort 1 (phase I and expansion cohorts phase) : non-small cell lung cancer without oncogenic addiction (EGFR/ALK/ROS/BRAF) that progresses after platinum-based chemotherapy and immunotherapy given as sequential or concomitant therapy
- Cohort 2 (expansion cohorts phase only) : bladder cancer that progresses after platinum-based therapy with or without maintenance with anti-PD-1/PD-L1
- Cohort 3 (expansion cohorts phase only): renal cell carcinoma in second-line therapy that progresses after a first-line administered according to the guidelines for the standard of care in renal cancer. First-line therapy could include an antiangiogenic agent, a tyrosine kinase inhibitor, alone or associated with immunotherapy (anti-PD-L1, anti-CTLA4)
- Cohort 4 (expansion cohorts phase only): head and neck carcinoma at the first line of locoregional or metastatic recurrence. The patient should not be eligible for surgery.

Study inclusion and exclusion criteria are detailed in Tables 1 and 2.

**Table 1:** Study inclusion criteria.

*Study inclusion criteria*
1. Male or female that must have signed a written informed consent prior to any study specific procedures
2. Aged ≥ 18 years at randomization
3. Performance status of 0 or 1 according to the WHO Easter Cooperative Oncology Group (ECOG)
4. Minimum 3 measureable lesions, with at least one lesion which cannot be irradiated for testing the abscopal effect and at least 2 irradiable lesions. Those lesions must be uni-dimensionally ≥ 10 mm considered as measurable according to RECIST v1.1.
5. Life expectancy > 6 months
6. At least one non-irradiated tumor site that can be biopsied for research purpose
7. Participant must have following advanced disease and must not be a candidate for other approved therapeutic regimen known to provide significant clinical benefit based on investigator judgement : - Cohort 1 (phase I and expansion cohorts phase) : non-small cell lung cancer without oncogenic addiction (EGFR/ALK/ROS/BRAF) which progress after platin based chemotherapy and immunotherapy given as sequential or concomitant therapy - Cohort 2 (expansion cohorts phase only) : bladder cancer which progress after platin based therapy with or without maintenance with anti PD-1/PD-L1 - Cohort 3 (expansion cohorts phase only) : renal cell carcinoma in second-line therapy which progress after a first line administered according to the guidelines for the standard of care in renal cancer. First line therapy could include antiangiogenic agent, tyrosine kinase inhibitor, alone or associated with immunotherapy (anti-PD-L1, anti-CTLA4) - Cohort 4 (expansion cohorts phase only) : head and neck carcinoma at the first line locoregional or metastatic recurrence. Patient should not be eligible to surgery.
8. Participants who received prior anti-PD-1/L1 therapy must fulfill the following requirements - Have achieved a complete response, partial response or stable disease and subsequently had disease progression while still on anti-PD-1/L1 therapy - Have received at least two doses of an approved anti-PD-1/L1 therapy (by any regulatory authority) - Have demonstrated disease progression as defined by RECIST v1.1
9. Adequate hematological, renal, metabolic and hepatic functions: - Hemoglobin ≥ 9 g/dl (participants may have received prior red blood cell [RBC] transfusion, if clinically indicated (but should be performed with 1 week delay between transfusion and validation of eligibility criteria); absolute neutrophil count (ANC) ≥ 1.5 G/l, platelet count ≥ 100 G/l, white blood cell count ≥ 2.5 G/l (or within local laboratory normal limits) and lymphocyte count ≥ 0.75 G/l -. Alkaline phosphatase (AP), alanine aminotransferase (ALT) and aspartate aminotransferase (ASP) ≤ 2.5 x upper limit of normality (ULN) (≤ 5 x ULN in case of extensive skeletal involvement for AP exclusively and ≤ 5 x ULN in case of liver metastasis for AST and ALT). - Total bilirubin ≤ 1.5 x ULN, (3 x ULN for gilbert disease) - Albumin ≥ 25 g/l. - Serum creatinine ≤ 1.5 x ULN or calculated creatinine clearance (CrCl) ≥ 30 ml/min (according to Cockcroft and Gault formula). - INR ≤ 1.5 x ULN - aPTT ≤ 1.5 X ULN - Serum calcium within normal laboratory ranges,
10. No prior or concurrent malignant disease if more recent than 3 years
11. At least three weeks since last chemotherapy, immunotherapy or any other pharmacological treatment and/or radiotherapy
12. At least 2 mesurable lesions should be able to receveid 3 X 8 Gy by SBRT according to the dose constraints to organs at risk (OAR) imposed by the protocol
13. In case of lesion previously treated by radiotherapy, the constraints on the organs at risk must be respected after having summed up the treatment plans

**Table 2:** Study exclusion criteria.

*Study exclusion criteria*
1. Evidence of symptomatic central nervous system (CNS) or leptomeningeal metastases
2. Women who are pregnant or breast feeding
3. Participation in a study involving a medical or therapeutic intervention in the last 30 days
4. Previous enrolment in the present study
5. Participant unable to follow and comply with the study procedures because of any geographical, familial, social or psychological reasons
6. Known hypersensitivity to any involved study drug or of its formulation components
7. History of severe allergic anaphylactic reactions to chimeric, human or humanized antibodies, or fusion proteins
8. Treatment with systemic immunosuppressive medications including, but not limited to, cyclophosphamide, azathioprine, methotrexate, thalidomide, and anti-TNF agents within 2 weeks prior to inclusion. Systemic corticosteroids is not allowed but at physiologic doses meaning less than 10 mg/day of prednisone or its equivalent. The use of inhaled corticosteroids and mineral corticoids is allowed
9. Major surgical procedure, open biopsy or significant traumatic injury within 28 days before inclusion
10. Any of the following cardiac criteria: - Congestive heart failure ≥ New York Heart Association (NYHA) class 2, - Unstable angina (angina symptoms at rest), new-onset angina (begun within the last 3 months), - Myocardial infarction less than 6 months before inclusion - Uncontrolled cardiac arrhythmias, - Known left ventricular ejection fraction (LVEF) < 50%
11. Individuals deprived of liberty or placed under legal guardianship, curatorship or judicial protection
12. Prior organ transplantation, including allogeneic stem cell transplantation
13. Known clinically significant liver disease, including, alcoholic, or other hepatitis, cirrhosis, fatty liver and inherited liver disease, active viral with positive viral DNA detection
14. History of intra-abdominal inflammatory process within the last 12 months such as, but not limited to, diverticulitis, peptic ulcer disease or colitis
15. History of autoimmune disease including, but not limited to systemic lupus erythematosus (SLE), Sjögren’s syndrome, glomerulonephritis, multiple sclerosis, rheumatoid arthritis, vasculitis, systemic immune activation, inflammatory bowel disease, vascular thrombosis associated with antiphospholipid syndrome, Wegener’s granulomatosis, Guillain-Barré syndrome, Bell’s palsy - Participants with a history of autoimmune-related hypothyroidism on a stable dose of thyroid replacement hormone are eligible - Participants with controlled Type I diabetes mellitus on a stable insulin regimen are eligible - Participants with eczema, psoriasis, lichen simplex chronicus, or vitiligo with only dermatologic manifestations < 10% of the skin (e.g., participants with psoriatic arthritis would be excluded) are eligible
16. History of idiopathic pulmonary fibrosis, organizing pneumonia (e.g. bronchiolitis obliterans), drug-induced pneumonitis, idiopathic pneumonitis, or evidence of active pneumonitis on screening chest CT scan. History of radiation pneumonitis in the radiation field (fibrosis) is permitted
17. Poorly controlled Type II diabetes mellitus defined as a screening fasting plasma glucose ≥ 160 mg/dL (or 8.8 mmol/L)
18. Severe infections within 2 weeks prior to inclusion, including but not limited to SARS-Cov-2 infection, hospitalization for complications of infection, bacteremia, or severe pneumonia
19. Received therapeutic oral or IV antibiotics within 2 weeks prior to inclusion. Participants receiving prophylactic antibiotics (e.g., for prevention of a urinary tract infection or chronic obstructive pulmonary disease) are eligible
20. Any contraindication to tumor biopsy
21. Participant with spinal cord compression not definitively treated with surgery and/or radiation or previously diagnosed and treated spinal cord compression without evidence that disease has been clinically stable for > 2 weeks prior to inclusion
22. Administration of a live, attenuated vaccine within 4 weeks before the start of study medication or anticipation that such a live attenuated vaccine will be required during the study. Influenza vaccination is allowed during influenza season only (approximatively October to March)
23. Patient with a known history of Human Immunodeficiency Virus (HIV) (HIV1/2 antibodies) or known acquired immunodeficiency syndrome (AIDS),
24. Patients with EBV infection (Positive EBV viral capsid antigen IgM test at screening)
25. Active tuberculosis
26. Concomitant use of prohibited concomitant therapy (cf paragraph 6.5.2.2) or anticipation that such concomitant medication/therapies will be required during the study
27. For NSCLC indication, patients with lymphoepithelioma-like carcinoma are excluded
28. Female patients who are pregnant or breastfeeding or male or female patients of reproductive potential who are not willing to employ effective birth control from screening to 5 months after the last dose of atezolizumab and/or tiragolumab and 6 months following the last dose of radiotherapy

### Investigational combination products

Atezolizumab is a humanized IgG1 Mab that targets PD-L1 and inhibits the interaction between PD-L1 and its receptors, PD-1 and B7-1 (also known as CD80), both of which function as inhibitory receptors expressed on T cells. Therapeutic blockade of PD-L1 binding by atezolizumab has been shown to enhance the magnitude and quality of tumor-specific T-cell responses. Atezolizumab has minimal binding to Fc receptors, thus eliminating detectable Fc-effector function and the associated antibody-mediated clearance of activated effector T cells.

Tiragolumab (formerly MTIG7192A) is a fully human IgG1/κ Mab derived in open monoclonal technology rats that binds to TIGIT and prevents its interaction with the poliovirus receptor. The recombinant antibody is produced in Chinese hamster ovary (CHO) cells and consists of two heavy chains (456 amino acid residues each) and two light chains (220 amino acid residues each). There are two N-linked glycosylation sites (Asn306) in the Fc domain. The predicted molecular weight of tiragolumab is 148,409 Da (peptide chains only, without heavy chain C-terminal lysine residue). By binding to TIGIT, tiragolumab blocks its interaction with a protein called CD155 (poliovirus receptor) that can suppress the body’s immune response. Therapeutic blockade of TIGIT by means of tiragolumab represents an attractive strategy for cancer therapy and is expected to enhance the magnitude and quality of the tumor-specific T-cell responses, which may result in improved meaningful anti-tumor activity when tiragolumab is used as a single agent or in combination with other ICIs.

### Investigational procedure

Each lesion will be treated with SBRT using 3 fractions of 8 Gy prescribed on the PTV (Planned Tumor Volume), with one fraction delivered every day, ideally Monday, Wednesday, and Friday, so the treatment period will therefore be five days. For technical reasons (breakdown, maintenance, etc.), two fractions will be delivered over two successive days, with a minimum delay of 24 hours between two fractions. A total treatment period of 5 days will be accepted.

Treatment must be delivered by a regular LINAC (Linear Accelerator) or MRI (Magnetic resonance Imaging) machine using the SBRT technique to all lesions and isocenters. Daily IGRT (Image-Guided Radiation Therapy) is required. If it’s possible, we encourage avoiding the VMAT (Volumetric Modulated Arc Therapy) technique to decrease the risk of lymphopenia, which may be associated with anti-tumoral immunity reduction. For this SBRT prescription condition, at least 95% of PTV will have to receive 100% of the prescribed dose (24 Gy).

Patients included in this study will present at least three lesions. In order to guide which lesions to irradiate, the following principles should be used:

- To irradiate at least two measurable lesions
- To let at least one measurable non-irradiated lesion to evaluate the abscopal effect. Of note, this non-irradiated lesion can be the one biopsied.
- To try to irradiate the majority of the lesions, however, it is mandatory to respect the constraints on OARs (organs at risk).

Dose constraints for OARs are defined in Table 3 according to Timmerman et al [21].

**Table 3:** Dose contraints to OARs

**OARs**	**Dmax (Gy)**	**Volume**
Optic nerve	19.5	V15 < 0.2 cc
Cochlea	20	
Brainstem	23	V18 < 1 cc
Spinal Cord	22	V18 < 0.25 cc; V11.1 < 1.2 cc
Cauda equina	24	V21.9 < 5 cc
Sacral plexus	24	V22.5 < 3 cc
Esophagus	27	V21 < 5 cc
Ipsilateral brachial plexus	24	V22.5 < 3 cc
Heart/pericardium	30	V24 < 15 cc
Great vessels	45	V39 < 10 cc
Trachea and Ipsilateral bronchus	30	V15 < 4 cc
Skin	24	V22.5 < 10 cc
Stomach	24	V21 < 10 cc
Duodenum	24	V15 < 5 cc
Jejunum / ileum	27	V16.2 < 5 cc
Colon	30	V20.4 < 20 cc
Kidneys (right and left) Rectum		V14 < 200 cc, V15 < 33%
Rectum		V20.4 < 20 cc
Bladder	30	V15 < 15 cc
Penil Bulb	30	V21.9 < 3 cc
Femoral Heads (right and left)	42	V21.9 < 10 cc
Renal Hilum/vascular trunk		V18.6 < 2/3 volume
Normal Lung (homolateral lung – ITV) (if one lung lesion)		V20 < 10%
Normal Lung (bilateral lung – ITV) (if several lung lesions)		V19 < 6.5%
Normal Liver (Liver – ITV)		V15 < 700 cc

### Statistical analyses

#### Phase I

Phase I will include a maximum of 18 patients (only non-small-cell lung cancer patients will be included in this step).

- 6 +/- 3 patients for the sequential scheme evaluation.
- 6 +/- 3 patients for the concomitant scheme evaluation.

As the safety of the anti-TIGIT and anti-PD-L1 combination has been previously validated, we focused the DLTs on radiation-induced toxicities. We chose to accept no more than 22% of radiation-induced toxicities, as patients will undergo additional chemotherapy-related toxicities.

#### Expansion cohorts phase

Depending on the results of toxicity from phase I, a sequential or concomitant scheme will be selected according to decision diagrams.

20 patients will be included in each cohort (including 6 to 9 patients from phase 1 for the lung cancer cohort). Each cohort will be analyzed independently.

20 patients in each cohort will allow for preliminary estimates of efficacy endpoints. A total sample of 18 subjects (assuming 10% of non-evaluable patients) provides a 95% CI with a precision of 22%, 23%, and 24% when the estimated rates of 6-month PFS (under binomial assumption and with no censored data) are 35%, 30%, and 45%, respectively, in head and neck, renal and lung cancers. For head and neck cancer, the 6-month PFS estimate of 35% was selected on the basis of the 6-month PFS rates achieved in Keynote-048 [8], 25% and 45% with pembrolizumab and pembrolizumab plus chemotherapy, respectively. For renal cancer, few data are available on immunotherapy efficacy after a first-line treatment consisting of an immune checkpoint inhibitor combination or an association of a tyrosine kinase inhibitor and immune checkpoint blockade. The chosen 6-month PFS rate is close to what has been observed in the CheckMate-025 [22] study with nivolumab as a second-line treatment. Lastly, for lung cancer, randomized clinical trials of second-line treatment were performed before the use of chemo-immunotherapy as the first-line standard of care [23–26]. In recent retrospective studies assessing chemotherapy in a second-line setting after immunotherapy, the 6-month PFS percentage approaches 40% similarly to the chosen rate [27, 28].

#### Definition of study population analysis

The safety population is defined by all patients who received at least 1 dose of immunotherapy combined with at least 1 radiation therapy session.

The modified-intention-to-treat (mITT) population is defined by all patients treated with the phase I recommended scheme who received at least one administration of each immunotherapy, the 3 radiotherapy fractions, and at least one tumor evaluation (CT-scan).

#### Statistical analysis of endpoints

The primary endpoint will be evaluated in the safety population. Secondary efficacy endpoints will be evaluated in the mITT population, and secondary safety endpoints in the safety population.

##### **Phase I**

The number of toxicities will be described by type and grade for each level (sequential and concomitant) according to the MedDRA classification. A listing of adverse events by patient and level will be provided.

##### Expansion cohort

Each cohort will be analyzed separately. PFS and OS rates (at 6 and 12 months) and median PFS and their respective 95% CI will be determined using the Kaplan-Meier method to take censored data into account. Toxicities will be described by type and grade according to the MedDRA classification. In the event a patient experiences several toxicities of the same type, only the highest grade will be retained for analysis. A listing of adverse events by patient will be provided. Analyses will be performed using SAS 9.4 or a later version.

### Interim analysis

An Independent Data Monitoring Committee (IDMC) will be established for the trial. This IDMC will meet after the inclusion of 10 patients with 5 weeks of follow-up in each cohort to evaluate toxicities, except for the lung cancer cohort, which will be evaluated during phase I. Toxicities will be described by grade and type of toxicity for each patient, focusing on the same DLT as described for phase I. The purpose of this interim analysis is to validate with the IDMC the safety of the combination of immunotherapy and radiation. Inclusion will be stopped while waiting for the IDMC meeting and decision. No interim analysis is planned for efficacy.

### Ethical and regulatory requirements

The study will be performed in accordance with ethical principles that have their origin in the Declaration of Helsinki, including Decree No. 2016 − 1537 of November 16, 2016 on research involving human subjects, and are consistent with ICH/Good Clinical Practice, and applicable regulatory requirements Patient Data Protection; in France, CNIL.

Before carrying out research on humans, the sponsor is required to submit the project to the opinion of one of the competent institutional ethical committees (Committee for the Protection of Persons: CPP) and to the regulatory authority (National Agency for the Safety of Medicines and Health Products: ANSM).

Prior to the implementation of the research on a person, the eligible subject will be fully informed by the investigator during the consultation, and after a period of reflection, a written informed consent form will be collected.

The information will also include information on data handling in accordance with the revised French Data Protection regulations including the European General Data Protection Regulation N° 2016/679.

## Discussion

Targeting immunomodulatory pathways beyond PD-1/PD-L1 is an emerging field in oncology. As LAG-3, TIM3, BTLA, and TIGIT belong to the co-inhibitory receptors, which are known to impair anti-tumor immunity [13]. Ongoing trials assess tiragolumab in the metastatic setting of non-small cell lung cancer (NCT04619797, NCT03977467, NCT05034055, NCT04958811, NCT04294810, NCT03563716), bladder cancer (NCT05645692) and head and neck cancer (NCT04665843), NCT05483400). To date, only one study, which focuses especially on non-small cell lung cancer, has integrated SBRT into the strategy (NCT05483400). Preclinical data has investigated the sequence of immunotherapy and SBRT, thus demonstrating that systemic immune checkpoint blockade given before irradiation impairs the anti-tumor immune response [29]. Consequently, we designed our administration schemes in order to maximize the systemic immune response.

Beyond safety, we aim to detect an activity signal of our combination. Secondary analyses will help to sustain emerging data that suggest the critical role of cytokine production in the tumor microenvironment [30–32]. The study is ongoing, and the first patient was included on December 5th, 2022.

### Electronic Supplementary Material

Below is the link to the electronic supplementary material

Additional File 1

## Data Availability

No data are available for this manuscript concerning the clinical trial protocol. Data may be shared later, after the final publication of results, with the permission of the pharmaceutical company.

## Abbreviations

APC: Antigen-presenting cells
CD8 T cell: CD8 T lymphocyte
CTLA4: Cytotoxic T-lymphocyte-associated protein 4
DC: Dendritic cell
DLN: Draining lymph node
DOR: Duration of response
HMGB1: High mobility group box 1
ICI: Immune checkpoint inhibitor
mITT: Modified-intention to treat
NPD: Non-progression duration
NPR: Non-progression rate
ORR: Overall response rate
OS: Overall survival
PD-1: Programmed death 1
PD-L1: Programmed death-ligand 1
PFS: Progression-free survival
PVR: Poliovirus receptor
RT: Radiotherapy
SBRT: Stereotactic body radiation therapy
TAA: Tumor-associated antigen
TLR4: Toll-like receptor 4
TNA: Tumor neoantigen
TIGIT: T cell immunoreceptor with Ig and ITIM domains
TME: Tumor microenvironment
TILs: Tumor-infiltrating lymphocytes

## References

[1] Vaddepally RK, Kharel P, Pandey R, Garje R, Chandra AB. Review of Indications of FDA-Approved Immune Checkpoint Inhibitors per NCCN Guidelines with the Level of Evidence. Cancers [Internet]. 2020 Mar 20 [cited 2022 Dec 6];12(3):738. Available from: https://www.mdpi.com/2072-6694/12/3/738. 10.3390/cancers12030738 PMC7140028 32245016

[2] Planchard D, Popat S, Kerr K, Novello S, Smit EF, Faivre-Finn C et al. Metastatic non-small cell lung cancer: ESMO Clinical Practice Guidelines for diagnosis, treatment and follow-up. Annals of Oncology [Internet]. 2018 Oct [cited 2022 Nov 28];29:iv192–237. Available from: https://linkinghub.elsevier.com/retrieve/pii/S0923753419317107. 10.1093/annonc/mdy275 30285222

[3] Escudier B, Porta C, Schmidinger M, Rioux-Leclercq N, Bex A, Khoo V et al. Renal cell carcinoma: ESMO Clinical Practice Guidelines for diagnosis, treatment and follow-up. Annals of Oncology [Internet]. 2019 May [cited 2022 Nov 28];30(5):706–20. Available from: https://linkinghub.elsevier.com/retrieve/pii/S0923753419311573. 10.1093/annonc/mdz056 30788497

[4] Powles T, Bellmunt J, Comperat E, De Santis M, Huddart R, Loriot Y et al. Bladder cancer: ESMO Clinical Practice Guideline for diagnosis, treatment and follow-up. Annals of Oncology [Internet]. 2022 Mar [cited 2022 Nov 28];33(3):244–58. Available from: https://linkinghub.elsevier.com/retrieve/pii/S0923753421048274. 10.1016/j.annonc.2021.11.012 34861372

[5] Machiels JP, René Leemans C, Golusinski W, Grau C, Licitra L, Gregoire V. Squamous cell carcinoma of the oral cavity, larynx, oropharynx and hypopharynx: EHNS–ESMO–ESTRO Clinical Practice Guidelines for diagnosis, treatment and follow-up. Annals of Oncology [Internet]. 2020 Nov [cited 2022 Nov 28];31(11):1462–75. Available from: https://linkinghub.elsevier.com/retrieve/pii/S092375342039949X. 10.1016/j.annonc.2020.07.011 33239190

[6] Gandhi L, Rodríguez-Abreu D, Gadgeel S, Esteban E, Felip E, De Angelis F et al. Pembrolizumab plus Chemotherapy in Metastatic Non–Small-Cell Lung Cancer. N Engl J Med [Internet]. 2018 May 31 [cited 2022 Dec 15];378(22):2078–92. Available from: http://www.nejm.org/doi/10.1056/NEJMoa1801005. 10.1056/NEJMoa1801005 29658856

[7] Paz-Ares L, Luft A, Vicente D, Tafreshi A, Gümüş M, Mazières J (2018). Pembrolizumab plus Chemotherapy for Squamous Non-Small-Cell Lung Cancer. N Engl J Med.

[8] Burtness B, Harrington KJ, Greil R, Soulières D, Tahara M, de Castro G (2019). Pembrolizumab alone or with chemotherapy versus cetuximab with chemotherapy for recurrent or metastatic squamous cell carcinoma of the head and neck (KEYNOTE-048): a randomised, open-label, phase 3 study. Lancet.

[9] Powles T, Park SH, Voog E, Caserta C, Valderrama BP, Gurney H (2020). Avelumab Maintenance Therapy for Advanced or Metastatic Urothelial Carcinoma. N Engl J Med.

[10] Bellmunt J, de Wit R, Vaughn DJ, Fradet Y, Lee JL, Fong L (2017). Pembrolizumab as Second-Line Therapy for Advanced Urothelial Carcinoma. N Engl J Med.

[11] Haslam A, Prasad V. Estimation of the Percentage of US Patients With Cancer Who Are Eligible for and Respond to Checkpoint Inhibitor Immunotherapy Drugs. JAMA Netw Open [Internet]. 2019 May 3 [cited 2022 Dec 6];2(5):e192535. Available from: http://jamanetworkopen.jamanetwork.com/article.aspx?doi=10.1001/jamanetworkopen.2019.2535. 10.1001/jamanetworkopen.2019.2535 PMC6503493 31050774

[12] Bruni D, Angell HK, Galon J (2020). The immune contexture and Immunoscore in cancer prognosis and therapeutic efficacy. Nat Rev Cancer.

[13] Kraehenbuehl L, Weng CH, Eghbali S, Wolchok JD, Merghoub T. Enhancing immunotherapy in cancer by targeting emerging immunomodulatory pathways. Nat Rev Clin Oncol [Internet]. 2022 Jan [cited 2022 Dec 6];19(1):37–50. Available from: https://www.nature.com/articles/s41571-021-00552-7. 10.1038/s41571-021-00552-7 34580473

[14] Rodriguez-Abreu D, Johnson ML, Hussein MA, Cobo M, Patel AJ, Secen NM et al. Primary analysis of a randomized, double-blind, phase II study of the anti-TIGIT antibody tiragolumab (tira) plus atezolizumab (atezo) versus placebo plus atezo as first-line (1L) treatment in patients with PD-L1-selected NSCLC (CITYSCAPE). JCO [Internet]. 2020 May 20 [cited 2022 Dec 6];38(15_suppl):9503–9503. 10.1200/JCO.2020.38.15_suppl.9503.

[15] Cho BC, Abreu DR, Hussein M, Cobo M, Patel AJ, Secen N (2022). Tiragolumab plus atezolizumab versus placebo plus atezolizumab as a first-line treatment for PD-L1-selected non-small-cell lung cancer (CITYSCAPE): primary and follow-up analyses of a randomised, double-blind, phase 2 study. Lancet Oncol.

[16] Apetoh L, Ghiringhelli F, Tesniere A, Obeid M, Ortiz C, Criollo A et al. Toll-like receptor 4–dependent contribution of the immune system to anticancer chemotherapy and radiotherapy. Nat Med [Internet]. 2007 Sep [cited 2022 Dec 6];13(9):1050–9. Available from: http://www.nature.com/articles/nm1622. 10.1038/nm1622 17704786

[17] Ngwa W, Irabor OC, Schoenfeld JD, Hesser J, Demaria S, Formenti SC. Using immunotherapy to boost the abscopal effect. Nat Rev Cancer [Internet]. 2018 May [cited 2022 Dec 9];18(5):313–22. Available from: http://www.nature.com/articles/nrc.2018.6. 10.1038/nrc.2018.6 PMC5912991 29449659

[18] Brooks ED, Chang JY. Time to abandon single-site irradiation for inducing abscopal effects. Nat Rev Clin Oncol [Internet]. 2019 Feb [cited 2022 Dec 9];16(2):123–35. Available from: http://www.nature.com/articles/s41571-018-0119-7. 10.1038/s41571-018-0119-7 30401936

[19] Luke JJ, Lemons JM, Karrison TG, Pitroda SP, Melotek JM, Zha Y et al. Safety and Clinical Activity of Pembrolizumab and Multisite Stereotactic Body Radiotherapy in Patients With Advanced Solid Tumors. JCO [Internet]. 2018 Jun 1 [cited 2022 Dec 9];36(16):1611–8. 10.1200/JCO.2017.76.2229. 10.1200/JCO.2017.76.2229 PMC5978468 29437535

[20] Grapin M, Richard C, Limagne E, Boidot R, Morgand V, Bertaut A et al. Optimized fractionated radiotherapy with anti-PD-L1 and anti-TIGIT: a promising new combination. j immunotherapy cancer [Internet]. 2019 Dec [cited 2022 Dec 20];7(1):160. Available from: https://jitc.bmj.com/lookup/doi/10.1186/s40425-019-0634-9. 10.1186/s40425-019-0634-9 PMC6593525 31238970

[21] Timmerman R. A Story of Hypofractionation and the Table on the Wall. International Journal of Radiation Oncology*Biology*Physics [Internet]. 2022 Jan [cited 2023 Feb 21];112(1):4–21. Available from: https://linkinghub.elsevier.com/retrieve/pii/S0360301621028315. 10.1016/j.ijrobp.2021.09.027 34919882

[22] Motzer RJ, Escudier B, McDermott DF, George S, Hammers HJ, Srinivas S et al. Nivolumab versus Everolimus in Advanced Renal-Cell Carcinoma. N Engl J Med [Internet]. 2015 Nov 5 [cited 2023 Jul 4];373(19):1803–13. Available from: http://www.nejm.org/doi/10.1056/NEJMoa1510665. 10.1056/NEJMoa1510665 PMC5719487 26406148

[23] Hanna N, Shepherd FA, Fossella FV, Pereira JR, De Marinis F, Von Pawel J et al. Randomized Phase III Trial of Pemetrexed Versus Docetaxel in Patients With Non-Small-Cell Lung Cancer Previously Treated With Chemotherapy. JCO [Internet]. 2023 May 20 [cited 2023 Jun 29];41(15):2682–90. 10.1200/JCO.22.02546. 10.1200/JCO.22.02546 37196429

[24] Shepherd FA, Dancey J, Ramlau R, Mattson K, Gralla R, O’Rourke M et al. Prospective Randomized Trial of Docetaxel Versus Best Supportive Care in Patients With Non–Small-Cell Lung Cancer Previously Treated With Platinum-Based Chemotherapy. JCO [Internet]. 2000 May 10 [cited 2023 Jun 29];18(10):2095–103. 10.1200/JCO.2000.18.10.2095. 10.1200/JCO.2000.18.10.2095 10811675

[25] Schuette W, Nagel S, Blankenburg T, Lautenschlaeger C, Hans K, Schmidt EW et al. Phase III Study of Second-Line Chemotherapy for Advanced Non–Small-Cell Lung Cancer With Weekly Compared With 3-Weekly Docetaxel. JCO [Internet]. 2005 Nov 20 [cited 2023 Jun 29];23(33):8389–95. 10.1200/JCO.2005.02.3739. 10.1200/JCO.2005.02.3739 16293869

[26] Moliner L, Spurgeon L, Califano R, Controversies. in NSCLC: which second-line strategy after chemo-immunotherapy? ESMO Open [Internet]. 2023 Apr [cited 2023 Jun 29];8(2):100879. Available from: https://linkinghub.elsevier.com/retrieve/pii/S2059702923000996. 10.1016/j.esmoop.2023.100879 PMC9958277 36791668

[27] Schvartsman G, Peng SA, Bis G, Lee JJ, Benveniste MFK, Zhang J et al. Response rates to single-agent chemotherapy after exposure to immune checkpoint inhibitors in advanced non-small cell lung cancer. Lung Cancer [Internet]. 2017 Oct [cited 2023 Jun 29];112:90–5. Available from: https://linkinghub.elsevier.com/retrieve/pii/S0169500217304336. 10.1016/j.lungcan.2017.07.034 29191606

[28] Park SE, Lee SH, Ahn JS, Ahn MJ, Park K, Sun JM. Increased Response Rates to Salvage Chemotherapy Administered after PD-1/PD-L1 Inhibitors in Patients with Non–Small Cell Lung Cancer. Journal of Thoracic Oncology [Internet]. 2018 Jan [cited 2023 Jun 29];13(1):106–11. Available from: https://linkinghub.elsevier.com/retrieve/pii/S1556086417328502. 10.1016/j.jtho.2017.10.011 29101058

[29] Wei J, Montalvo-Ortiz W, Yu L, Krasco A, Ebstein S, Cortez C et al. Sequence of αPD-1 relative to local tumor irradiation determines the induction of abscopal antitumor immune responses. Sci Immunol [Internet]. 2021 Apr 2 [cited 2023 Jun 29];6(58):eabg0117. Available from: https://www.science.org/doi/10.1126/sciimmunol.abg0117. 10.1126/sciimmunol.abg0117 33837124

[30] Roussot N, Ghiringhelli F, Rébé C (2022). Tumor Immunogenic Cell Death as a Mediator of Intratumor CD8 T-Cell Recruitment. Cells.

[31] Fumet JD, Limagne E, Thibaudin M, Ghiringhelli F (2020). Immunogenic Cell Death and Elimination of Immunosuppressive Cells: A Double-Edged Sword of Chemotherapy. Cancers (Basel).

[32] Limagne E, Nuttin L, Thibaudin M, Jacquin E, Aucagne R, Bon M (2022). MEK inhibition overcomes chemoimmunotherapy resistance by inducing CXCL10 in cancer cells. Cancer Cell.

