# Supplementary material for: A phase I study of the combination of atezolizumab, tiragolumab, and stereotactic body radiation therapy in patients with metastatic multiorgan cancer
Source: BMC Cancer. 2023 Nov 9;23:1080. doi: 10.1186/s12885-023-11534-6 (PMC10633948; doi:10.1186/s12885-023-11534-6)
Supplement: Supplementary file 1 — Additional File 1 [file 12885_2023_11534_MOESM1_ESM.pdf]

Supplementary Appendix: management of toxicities.

Management Guidelines for Pulmonary Events, Including Pneumonitis

Event	Management
**Pulmonary event, Grade 1**	Continue tiragolumab and atezolizumab and monitor closely.<br>Re-evaluate on serial imaging.<br>Consider patient referral to pulmonary specialist.
**Pulmonary event, Grade 2**	Withhold tiragolumab and atezolizumab for up to 12 weeks after event onset. [a]<br>Refer patient to pulmonary and infectious disease specialists and consider bronchoscopy or BAL.<br>Initiate treatment with corticosteroids equivalent to 1-2 mg/kg/day oral prednisone.<br>If event resolves to Grade 1 or better, resume tiragolumab and atezolizumab. [b]<br>If event does not resolve to Grade 1 or better while withholding tiragolumab and atezolizumab, permanently discontinue tiragolumab and atezolizumab and contact Medical Monitor. [c]<br>For recurrent events, treat as a Grade 3 or 4 event.
**Pulmonary event, Grade 3 or 4**	Permanently discontinue tiragolumab and atezolizumab and contact Medical Monitor. [c]<br>Bronchoscopy or BAL is recommended.<br>Initiate treatment with corticosteroids equivalent to 1-2 mg/kg/day oral prednisone.<br>If event does not improve within 48 hours after initiating corticosteroids, consider adding an immunosuppressive agent.<br>If event resolves to Grade 1 or better, taper corticosteroids over ≥ 1 month.

BAL : bronchoscopic alveolar lavage.

[a] Tiragolumab and atezolizumab may be withheld for a longer period of time (i.e., > 12 weeks after event onset) to allow for corticosteroids (if initiated) to be reduced to the equivalent of ≤ 10 mg/day oral prednisone. The acceptable length of the extended period of time must be agreed upon by the investigator and the Medical Monitor.

[b] If corticosteroids have been initiated, they must be tapered over ≥ 1 month to the equivalent of ≤ 10 mg/day oral prednisone before tiragolumab and atezolizumab can be resumed.

[c] Resumption of tiragolumab and atezolizumab may be considered in patients who are deriving benefit and have fully recovered from the immune-mediated event. Patients can be rechallenged with tiragolumab and atezolizumab only after approval has been documented by both the investigator (or an appropriate delegate) and the Medical Monitor.

Management Guidelines for Hepatic Events

Event	Management
**Hepatic event, Grade 1**	Continue tiragolumab and atezolizumab.<br>Monitor LFTs until values resolve to within normal limits or to baseline values.
**Hepatic event, Grade 2**	**All events:**<br>Monitor LFTs more frequently until return to baseline values.<br>**Events of > 5 days' duration:**<br>Withhold tiragolumab and atezolizumab for up to 12 weeks after event onset. [a]<br>Initiate treatment with corticosteroids equivalent to 1-2 mg/kg/day oral prednisone.<br>If event resolves to Grade 1 or better, resume tiragolumab and atezolizumab. [b]<br>If event does not resolve to Grade 1 or better while withholding tiragolumab and atezolizumab, permanently discontinue tiragolumab and atezolizumab and contact Medical Monitor. [c]
**Hepatic event, Grade 3 or 4**	Permanently discontinue tiragolumab and atezolizumab and contact Medical Monitor. [c]<br>Consider patient referral to gastrointestinal specialist for evaluation and liver biopsy to establish etiology of hepatic injury.<br>Initiate treatment with corticosteroids equivalent to 1-2 mg/kg/day oral prednisone.<br>If event does not improve within 48 hours after initiating corticosteroids, consider adding an immunosuppressive agent. If event resolves to Grade 1 or better, taper corticosteroids over ≥ 1 month.

LFT = liver function test.

[a] Tiragolumab and atezolizumab may be withheld for a longer period of time (i.e., > 12 weeks after event onset) to allow for corticosteroids (if initiated) to be reduced to the equivalent of 10 mg/day oral prednisone. The acceptable length of the extended period of time must be agreed upon by the investigator and the Medical Monitor.

[b] If corticosteroids have been initiated, they must be tapered over ≥ 1 month to the equivalent of ≤ 10 mg/day oral prednisone before tiragolumab and atezolizumab can be resumed.

[c] Resumption of tiragolumab and atezolizumab may be considered in patients who are deriving benefit and have fully recovered from the immune-mediated event. Patients can be rechallenged with tiragolumab and atezolizumab only after approval has been documented by both the investigator (or an appropriate delegate) and the Medical Monitor.

Management guidelines for diarrhea or colitis:

Event	Management
**Diarrhea or colitis, Grade 1**	Continue tiragolumab and atezolizumab.<br>Initiate symptomatic treatment.<br>Endoscopy is recommended if symptoms persist for > 7 days.<br>Monitor closely.
**Diarrhea or colitis, Grade 2**	Withhold tiragolumab and atezolizumab for up to 12 weeks after event onset. a<br>Initiate symptomatic treatment.<br>Patient referral to GI specialist is recommended.<br>For recurrent events or events that persist > 5 days, initiate treatment with corticosteroids equivalent to 1-2 mg/kg/day oral prednisone.<br>If event resolves to Grade 1 or better, resume tiragolumab and atezolizumab. b<br>If event does not resolve to Grade 1 or better while withholding tiragolumab and atezolizumab, permanently discontinue tiragolumab and atezolizumab and contact Medical Monitor. c
**Diarrhea or colitis, Grade 3**	• Withhold tiragolumab and atezolizumab for up to 12 weeks after event onset. Refer patient to GI specialist for evaluation and confirmatory biopsy.<br>• Initiate treatment with corticosteroids equivalent to 1-2 mg/kg/day IV methylprednisolone and convert to 1-2 mg/kg/day oral prednisone or equivalent upon improvement.<br>• If event resolves to Grade 1 or better, resume tiragolumab and atezolizumab. b If event does not resolve to Grade 1 or better while withholding tiragolumab and atezolizumab, permanently discontinue tiragolumab and atezolizumab and contact Medical Monitor. c
**Diarrhea or colitis, Grade 4**	• Permanently discontinue tiragolumab and atezolizumab and contact Medical Monitor. c Refer patient to GI specialist for evaluation and confirmation biopsy.<br>• Initiate treatment with corticosteroids equivalent to 1-2 mg/kg/day IV methylprednisolone and convert to 1-2 mg/kg/day oral prednisone or equivalent upon improvement.<br>• If event does not improve within 48 hours after initiating corticosteroids, consider adding an immunosuppressive agent.<br>If event resolves to Grade 1 or better, taper corticosteroids over ≥ 1 month.

GI = gastrointestinal.

a Tiragolumab and atezolizumab may be withheld for a longer period of time (i.e., > 12 weeks after event onset) to allow for corticosteroids (if initiated) to be reduced to the equivalent of ≤ 10 mg/day oral prednisone. The acceptable length of the extended period of time must be agreed upon by the investigator and the Medical Monitor. b If corticosteroids have been initiated, they must be tapered over ≥ 1 month to the equivalent of ≤ 10 mg/day oral prednisone before tiragolumab and atezolizumab can be resumed. c Resumption of tiragolumab and atezolizumab may be considered in patients who are deriving benefit and have fully recovered from the immune-mediated event. Patients can be rechallenged with tiragolumab and atezolizumab only after approval has been documented by both the investigator (or an appropriate delegate) and the Medical Monitor.

Management Guidelines for Endocrine Events

Event	Management
**Asymptomatic hypothyroidism**	Continue tiragolumab and atezolizumab.<br>Initiate treatment with thyroid replacement hormone.<br>Monitor TSH weekly.
**Symptomatic hypothyroidism**	Withhold tiragolumab and atezolizumab.<br>Initiate treatment with thyroid replacement hormone.<br>Monitor TSH weekly.<br>Consider patient referral to endocrinologist.<br>Resume tiragolumab and atezolizumab when symptoms are controlled and thyroid function is improving.
**Asymptomatic hyperthyroidism**	TSH $\geq$ 0.1 mU/L and < 0.5 mU/L:<br>Continue tiragolumab and atezolizumab.<br>Monitor TSH every 4 weeks.<br>TSH < 0.1 mU/L:<br>Follow guidelines for symptomatic hyperthyroidism.
**Symptomatic hyperthyroidism**	Withhold tiragolumab and atezolizumab.<br>Initiate treatment with anti-thyroid drug such as methimazole or carbimazole as needed.<br>Consider patient referral to endocrinologist.<br>Resume tiragolumab and atezolizumab when symptoms are controlled and thyroid function is improving.<br>Permanently discontinue tiragolumab and atezolizumab and contact Medical Monitor for life-threatening immune-mediated hyperthyroidism. c
**Symptomatic adrenal insufficiency, Grades 2-4**	Withhold tiragolumab and atezolizumab for up to 12 weeks after event onset. a<br>Refer patient to endocrinologist.<br>Perform appropriate imaging.<br>Initiate treatment with corticosteroids equivalent to 1-2 mg/kg/day IV methylprednisolone and convert to 1-2 mg/kg/day oral prednisone or equivalent upon improvement.<br>If event resolves to Grade 1 or better and patient is stable on replacement therapy, resume tiragolumab and atezolizumab. b<br>If event does not resolve to Grade 1 or better or patient is not stable on replacement therapy while withholding tiragolumab and atezolizumab, permanently discontinue tiragolumab and atezolizumab and contact Medical Monitor. c
**Hyperglycemia, Grade 1 or 2**	Continue tiragolumab and atezolizumab.<br>Investigate for diabetes. If patient has Type 1 diabetes, treat as a Grade 3 event. If patient does not have Type 1 diabetes, treat as per institutional guidelines.<br>Monitor for glucose control.
**Hyperglycemia, Grade 3 or 4**	Withhold tiragolumab and atezolizumab.<br>Initiate treatment with insulin.<br>Monitor for glucose control.<br>Resume tiragolumab and atezolizumab when symptoms resolve and glucose levels are stable.

**Hypophysitis (panhypopituitarism), Grade 2 or 3**	Withhold tiragolumab and atezolizumab for up to 12 weeks after event onset. a<br>Refer patient to endocrinologist.<br>Perform brain MRI (pituitary protocol).<br>Initiate treatment with corticosteroids equivalent to 1□2 mg/kg/day IV methylprednisolone and convert to 1□2 mg/kg/day oral prednisone or equivalent upon improvement.<br>Initiate hormone replacement if clinically indicated.<br>If event resolves to Grade 1 or better, resume tiragolumab and atezolizumab. b<br>If event does not resolve to Grade 1 or better while withholding tiragolumab and atezolizumab permanently discontinue tiragolumab and atezolizumab. c<br>For recurrent hypophysitis, treat as a Grade 4 event.
**Hypophysitis (panhypopituitarism), Grade 4**	Permanently discontinue tiragolumab and atezolizumab and contact Medical Monitor. c<br>Refer patient to endocrinologist.<br>Perform brain MRI (pituitary protocol).<br>Initiate treatment with corticosteroids equivalent to 1-2 mg/kg/day IV methylprednisolone and convert to 1-2 mg/kg/day oral prednisone or equivalent upon improvement.<br>Initiate hormone replacement if clinically indicated.

MRI = magnetic resonance imaging; TSH = thyroid-stimulating hormone. a Tiragolumab and atezolizumab may be withheld for a longer period of time (i.e., > 12 weeks after event onset) to allow for corticosteroids (if initiated) to be reduced to the equivalent of ≤ 10 mg/day oral prednisone. The acceptable length of the extended period of time must be agreed upon by the investigator and the Medical Monitor.

b If corticosteroids have been initiated, they must be tapered over ≥ 1 month to the equivalent of ≤ 10 mg/day oral prednisone before tiragolumab and atezolizumab can be resumed. c Resumption of tiragolumab and atezolizumab may be considered in patients who are deriving benefit and have fully recovered from the immune-mediated event. Patients can be re-challenged with tiragolumab and atezolizumab only after approval has been documented by both the investigator (or an appropriate delegate) and the Medical Monitor.

Management Guidelines for Ocular Events

Event	Management
**Ocular event, Grade 1**	Continue tiragolumab and atezolizumab.<br>Patient referral to ophthalmologist is strongly recommended.<br>Initiate treatment with topical corticosteroid eye drops and topical immunosuppressive therapy.<br>If symptoms persist, treat as a Grade 2 event.
**Ocular event, Grade 2**	Withhold tiragolumab and atezolizumab for up to 12 weeks after event onset. a<br>Patient referral to ophthalmologist is strongly recommended.<br>Initiate treatment with topical corticosteroid eye drops and topical immunosuppressive therapy.
	If event resolves to Grade 1 or better, resume tiragolumab and atezolizumab. b<br>If event does not resolve to Grade 1 or better while withholding tiragolumab and atezolizumab, permanently discontinue tiragolumab and atezolizumab and contact Medical Monitor. c
**Ocular event, Grade 3 or 4**	Permanently discontinue tiragolumab and atezolizumab and contact Medical Monitor. c<br>Refer patient to ophthalmologist.<br>Initiate treatment with corticosteroids equivalent to 1-2 mg/kg/day oral prednisone.<br>If event resolves to Grade 1 or better, taper corticosteroids over ≥ 1 month.

a Tiragolumab and atezolizumab may be withheld for a longer period of time (i.e., > 12 weeks after event onset) to allow for corticosteroids (if initiated) to be reduced to the equivalent of ≤ 10 mg/day oral prednisone. The acceptable length of the extended period of time must be agreed upon by the investigator and the Medical Monitor.

b If corticosteroids have been initiated, they must be tapered over ≥ 1 month to the equivalent of ≤ 10 mg/day oral prednisone before tiragolumab and atezolizumab can be resumed.

c Resumption of tiragolumab and atezolizumab may be considered in patients who are deriving benefit and have fully recovered from the immune-mediated event. Patients can be re-challenged with tiragolumab and atezolizumab only after approval has been documented by both the investigator (or an appropriate delegate) and the Medical Monitor.

Management Guidelines for Immune-Mediated Myocarditis

Event	Management
**Immune-mediated myocarditis, Grade 2**	Withhold tiragolumab and atezolizumab for up to 12 weeks after event onset [a] and contact Medical Monitor. Refer patient to cardiologist.<br>Initiate treatment as per institutional guidelines and consider antiarrhythmic drugs, temporary pacemaker, ECMO, or VAD as appropriate.<br>Consider treatment with corticosteroids equivalent to 1-2 mg/kg/day IV methylprednisolone and convert to 1-2 mg/kg/day oral prednisone or equivalent upon improvement.<br>If event resolves to Grade 1 or better, resume tiragolumab and atezolizumab. [b] If event does not resolve to Grade 1 or better while withholding tiragolumab and atezolizumab, permanently discontinue tiragolumab and atezolizumab and contact Medical Monitor. [c]
**Immune-mediated myocarditis, Grades 3 and 4**	Permanently discontinue tiragolumab and atezolizumab and contact Medical Monitor. [c]<br>Refer patient to cardiologist.<br>Initiate treatment as per institutional guidelines and consider antiarrhythmic drugs, temporary pacemaker, ECMO, or VAD as appropriate.<br>Initiate treatment with corticosteroids equivalent to 1-2 mg/kg/day IV methylprednisolone and convert to 1-2 mg/kg/day oral prednisone or equivalent upon improvement.<br>If event does not improve within 48 hours after initiating corticosteroids, consider adding an immunosuppressive agent.<br>If event resolves to Grade 1 or better, taper corticosteroids over ≥ 1 month.

ECMO = extracorporeal membrane oxygenation; VAD = ventricular assist device.

a [a] Tiragolumab and atezolizumab may be withheld for a longer period of time (i.e., > 12 weeks after event onset) to allow for corticosteroids (if initiated) to be reduced to the equivalent of ≤ 10 mg/day oral prednisone. The acceptable length of the extended period of time must be agreed upon by the investigator and the Medical Monitor. [b] If corticosteroids have been initiated, they must be tapered over ≥ 1 month to the equivalent of ≤ 10 mg/day oral prednisone before tiragolumab and atezolizumab can be resumed. [c] Resumption of tiragolumab and atezolizumab may be considered in patients who are deriving benefit and have fully recovered from the immune-mediated event. Patients can be re-challenged with tiragolumab and atezolizumab only after approval has been documented by both the investigator (or an appropriate delegate) and the Medical Monitor.

Management Guidelines for Infusion-Related Reactions

Event	Management
**IRR, Grade 1**	Reduce infusion rate to half the rate being given at the time of event onset.<br>After the event has resolved, the investigator should wait for 30 minutes while delivering the infusion at the reduced rate.<br>If the infusion is tolerated at the reduced rate for 30 minutes after symptoms have resolved, the infusion rate may be increased to the original rate.
**IRR, Grade 2**	Interrupt infusion.<br>Administer aggressive symptomatic treatment (e.g., oral or IV antihistamine, antipyretic medication, glucocorticoids, epinephrine, bronchodilators, oxygen, IV fluids).<br>After symptoms have resolved to baseline, resume infusion at half the rate being given at the time of event onset.
	For subsequent infusions, consider administration of oral premedication with antihistamines, antipyretic medications, and/or analgesics and monitor closely for IRRs.
**IRR, Grade 3 or 4**	Stop infusion.<br>Administer aggressive symptomatic treatment (e.g., oral or IV antihistamine, antipyretic medication, glucocorticoids, epinephrine, bronchodilators, oxygen, IV fluids).<br>Permanently discontinue tiragolumab or atezolizumab and contact Medical Monitor. [a]

IRR = infusion-related reaction.

[a] Resumption of tiragolumab or atezolizumab may be considered in patients who are deriving benefit and have fully recovered from the event. Patients can be re-challenged with tiragolumab or atezolizumab only after approval has been documented by both the investigator (or an appropriate delegate) and the Medical Monitor.

Management Guidelines for Cytokine-Release Syndrome

Event	Management
<u>Grade 1</u> [a]<br>**Fever [b] with or without constitutional symptoms**	• Immediately interrupt infusion.<br>• Upon symptom resolution, wait for 30 minutes and then restart infusion at half the rate being given at the time of event onset.<br>• If the infusion is tolerated at the reduced rate for 30 minutes, the infusion rate may be increased to the original rate.<br>• If symptoms recur, discontinue infusion of this dose.<br>• Administer symptomatic treatment, [c] including maintenance of IV fluids for hydration.<br>• In case of rapid decline or prolonged CRS (> 2 days) or in patients with significant symptoms and/or comorbidities, consider managing as per Grade 2.<br>• For subsequent infusions, consider administration of oral premedication with antihistamines, anti-pyretics, and/or analgesics, and monitor closely for CRS.
<u>Grade 2</u> [a]<br>**Fever [b] with at least one of the following:**	• Immediately interrupt infusion.<br>• Upon symptom resolution, wait for 30 minutes and then restart infusion at half the rate being given at the time of event onset.<br>• If symptoms recur, discontinue infusion of this dose.<br>• Administer symptomatic treatment. [c]

**Hypotension not requiring vasopressors**<br>**Hypoxia requiring lowflow oxygen [d] by nasal cannula or blow-by**	• For hypotension, administer IV fluid bolus as needed.<br>• Monitor cardiopulmonary and other organ function closely (in the ICU, if appropriate). Administer IV fluids as clinically indicated, and manage constitutional symptoms and organ toxicities as per institutional practice.<br>• Rule out other inflammatory conditions that can mimic CRS (e.g., sepsis). If no improvement within 24 hours, initiate workup and assess for signs and symptoms of HLH or MAS as described in this appendix.<br>• Consider IV corticosteroids (e.g., methylprednisolone 2 mg/kg/day or dexamethasone 10 mg every 6 hours).<br>• Consider anti-cytokine therapy.<br>• Consider hospitalization until complete resolution of symptoms. If no improvement within 24 hours, manage as per Grade 3, that is, hospitalize patient (monitoring in the ICU is recommended), permanently discontinue tiragolumab and atezolizumab, and contact Medical Monitor.<br>• If symptoms resolve to Grade 1 or better for 3 consecutive days, the next dose of tiragolumab and atezolizumab may be administered. For subsequent infusions, consider administration of oral premedication with antihistamines, antipyretics, and/or analgesics and monitor closely for CRS.<br>• If symptoms do not resolve to Grade 1 or better for 3 consecutive days, contact Medical Monitor.

**<u>Grade 3</u>** [a]<br>**Fever** [b] **with at least one of the following:**<br>**Hypotension requiring a vasopressor (with or without vasopressin)**<br>**Hypoxia requiring high-flow oxygen** [d] **by nasal cannula, face mask, non-rebreather mask, or Venturi mask**	• Permanently discontinue tiragolumab and atezolizumab and contact Medical Monitor. [e]<br>• Administer symptomatic treatment. [c]<br>• For hypotension, administer IV fluid bolus and vasopressor as needed.<br>• Monitor cardiopulmonary and other organ function closely; monitoring in the ICU is recommended. Administer IV fluids as clinically indicated, and manage constitutional symptoms and organ toxicities as per institutional practice. Rule out other inflammatory conditions that can mimic CRS (e.g., sepsis). If no improvement within 24 hours, initiate workup and assess for signs and symptoms of HLH or MAS as described in this appendix.<br>• Administer IV corticosteroids (e.g., methylprednisolone 2 mg/kg/day or dexamethasone 10 mg every 6 hours).<br>• Consider anti-cytokine therapy.<br>• Hospitalize patient until complete resolution of symptoms. If no improvement within 24 hours, manage as per Grade 4, that is, admit patient to ICU and initiate hemodynamic monitoring, mechanical ventilation, and/or IV fluids and vasopressors as needed; for patients who are refractory to anti-cytokine therapy, experimental treatments may be considered at the discretion of the investigator and in consultation with the Medical Monitor.
**<u>Grade 4</u>** [a]<br>**Fever** [b] **with at least one of the following:**<br>**Hypotension requiring multiple vasopressors (excluding vasopressin)**<br>**Hypoxia requiring oxygen by positive pressure (e.g., CPAP, BiPAP, intubation and mechanical ventilation)**	• Permanently discontinue tiragolumab and atezolizumab and contact Medical Monitor. [e]<br>• Administer symptomatic treatment. [c]<br>• Admit patient to ICU and initiate hemodynamic monitoring, mechanical ventilation, and/or IV fluids and vasopressors as needed. Monitor other organ function closely. Manage constitutional symptoms and organ toxicities as per institutional practice.<br>• Rule out other inflammatory conditions that can mimic CRS (e.g., sepsis). If no improvement within 24 hours, initiate workup and assess for signs and symptoms of HLH or MAS as described in this appendix.<br>• Administer IV corticosteroids (e.g., methylprednisolone 2 mg/kg/day or dexamethasone 10 mg every 6 hours).
	• Consider anti-cytokine therapy. For patients who are refractory to anti-cytokine therapy, experimental treatments may be considered at the discretion of the investigator and in consultation with the Medical Monitor.<br>• Hospitalize patient until complete resolution of symptoms.

ASTCT= American Society for Transplantation and Cellular Therapy; BiPAP = bi-level positive airway pressure; CAR = chimeric antigen receptor; CPAP = continuous positive airway pressure; CRS = cytokine-release syndrome; CTCAE = Common Terminology Criteria for Adverse Events; eCRF = electronic Case Report Form; HLH = hemophagocytic lymphohistiocytosis; ICU = intensive care unit; MAS = macrophage activation syndrome; NCCN = National Cancer Comprehensive Network; NCI = National Cancer Institute.

The management guidelines have been adapted from NCCN guidelines for management of CAR T-cell-related toxicities (Version 2.2019). [a] Grading system for management guidelines is based on the ASTCT CRS consensus grading scale. NCI CTCAE v5.0 and the ASTCT CRS consensus grading scale should be used when reporting severity of CRS on the Adverse Event eCRF. NCI CTCAE v5.0 should be used when reporting severity of organ toxicities associated with CRS on the dedicated Cytokine-Release Syndrome eCRF. Organ toxicities associated with CRS should not influence overall CRS grading.

[b] Fever is defined as temperature ≥ 38°C not attributable to any other cause. In patients who develop CRS and then receive anti-pyretic, anti-cytokine, or corticosteroid therapy, fever is no longer required when subsequently determining event severity (grade). In this case, the grade is driven by the presence of hypotension and/or hypoxia. [c] Symptomatic treatment may include oral or IV antihistamines, anti-pyretics, analgesics, bronchodilators, and/or oxygen. For bronchospasm, urticaria, or dyspnea, additional treatment may be administered as per institutional practice. [d] Low flow is defined as oxygen delivered at ≤ 6 L/min, and high flow is defined as oxygen delivered at > 6 L/min. [e] Resumption of tiragolumab and atezolizumab may be considered in patients who are deriving benefit and have fully recovered from the event. Patients can be re-challenged with tiragolumab and atezolizumab only after approval has been documented by both the investigator (or an appropriate delegate) and the Medical Monitor. For subsequent infusions, administer oral premedication with antihistamines, anti-pyretics, and/or analgesics, and monitor closely for CRS. Premedication with corticosteroids and extending the infusion time may also be considered after consulting the Medical Monitor and considering the benefit□risk ratio.

Management Guidelines for Pancreatic Events, Including Pancreatitis

Event	Management
**Amylase and/or lipase elevation, Grade 2**	• **Amylase and/or lipase > 1.5-2.0 x ULN:**<br>• Continue tiragolumab and atezolizumab. Monitor amylase and lipase weekly.<br>• For prolonged elevation (e.g., > 3 weeks), consider treatment with corticosteroids equivalent to 10 mg/day oral prednisone.<br>• **Asymptomatic with amylase and/or lipase > 2.0-5.0 x ULN:**<br>• Treat as a Grade 3 event.
**Amylase and/or lipase elevation, Grade 3 or 4**	Withhold tiragolumab and atezolizumab for up to 12 weeks after event onset. [a]<br>Refer patient to gastrointestinal specialist.<br>Monitor amylase and lipase every other day.<br>If no improvement, consider treatment with corticosteroids equivalent to 1-2 mg/kg/day oral prednisone.<br>If event resolves to Grade 1 or better, resume tiragolumab and atezolizumab. [b]<br>If event does not resolve to Grade 1 or better while withholding tiragolumab and atezolizumab, permanently discontinue tiragolumab and atezolizumab and contact Medical Monitor. [c]<br>For recurrent events, permanently discontinue tiragolumab and atezolizumab and contact Medical Monitor. [c]
**Immune-mediated pancreatitis, Grade 2 or 3**	Withhold tiragolumab and atezolizumab for up to 12 weeks after event onset. [a]<br>Refer patient to GI specialist.<br>Initiate treatment with corticosteroids equivalent to 1-2 mg/kg/day IV methylprednisolone and convert to 1-2 mg/kg/day oral prednisone or equivalent upon improvement.<br>If event resolves to Grade 1 or better, resume tiragolumab and atezolizumab. [b]<br>If event does not resolve to Grade 1 or better while withholding tiragolumab and atezolizumab, permanently discontinue tiragolumab and atezolizumab and contact Medical Monitor. [c]<br>For recurrent events, permanently discontinue tiragolumab and atezolizumab and contact Medical Monitor. [c]
**Immune-mediated pancreatitis, Grade 4**	Permanently discontinue tiragolumab and atezolizumab and contact Medical Monitor. [c]<br>Refer patient to gastrointestinal specialist.<br>Initiate treatment with corticosteroids equivalent to 1-2 mg/kg/day IV methylprednisolone and convert to 1-2 mg/kg/day oral prednisone or equivalent upon improvement.<br>If event does not improve within 48 hours after initiating corticosteroids, consider adding an immunosuppressive agent.<br>If event resolves to Grade 1 or better, taper corticosteroids over ≥ 1 month.

[a] Tiragolumab and atezolizumab may be withheld for a longer period of time (i.e., > 12 weeks after event onset) to allow for corticosteroids (if initiated) to be reduced to the equivalent of ≤ 10 mg/day oral prednisone. The acceptable length of the extended period of time must be agreed upon by the

investigator and the Medical Monitor. [b] If corticosteroids have been initiated, they must be tapered over ≥ 1 month to the equivalent of ≤10 mg/day oral prednisone before tiragolumab and atezolizumab can be resumed. [c] Resumption of tiragolumab and atezolizumab may be considered in patients who are deriving benefit and have fully recovered from the immune-mediated event. Patients can be re-challenged with tiragolumab and atezolizumab only after approval has been documented by both the investigator (or an appropriate delegate) and the Medical Monitor.

Management Guidelines for Dermatologic Events

Event	Management
**Dermatologic event, Grade 1**	• Continue tiragolumab and atezolizumab.<br>• Consider treatment with topical corticosteroids and/or other symptomatic therapy (e.g., antihistamines).
**Dermatologic event, Grade 2**	• Continue tiragolumab and atezolizumab.<br>• Consider patient referral to dermatologist for evaluation and, if indicated, biopsy.<br>• Initiate treatment with topical corticosteroids.<br>• Consider treatment with higher-potency topical corticosteroids if event does not improve.
**Dermatologic event, Grade 3**	• Withhold tiragolumab and atezolizumab for up to 12 weeks after event onset. [a]<br>• Refer patient to dermatologist for evaluation and, if indicated, biopsy.<br>• Initiate treatment with corticosteroids equivalent to 10 mg/day oral prednisone, increasing dose to 1-2 mg/kg/day if event does not improve within 48-72 hours.<br>• If event resolves to Grade 1 or better, resume tiragolumab and atezolizumab. [b]<br>• If event does not resolve to Grade 1 or better while withholding tiragolumab and atezolizumab, permanently discontinue tiragolumab and atezolizumab and contact Medical Monitor. [c]
**Dermatologic event, Grade 4**	• Permanently discontinue tiragolumab and atezolizumab and contact Medical Monitor. [c]
**Stevens-Johnson syndrome or toxic epidermal necrolysis, (any grade)**	• **Additional guidance for Stevens-Johnson syndrome or toxic epidermal necrolysis:**<br>• Withhold tiragolumab and atezolizumab for suspected Stevens-Johnson syndrome or toxic epidermal necrolysis.<br>• Confirm diagnosis by referring patient to a specialist (dermatologist, ophthalmologist, or urologist as relevant) for evaluation and, if indicated, biopsy.<br>• Follow the applicable treatment and management guidelines above.<br>• If Stevens-Johnson syndrome or toxic epidermal necrolysis is confirmed, permanently discontinue tiragolumab and atezolizumab.

[a] Tiragolumab and atezolizumab may be withheld for a longer period of time i.e., > 12 weeks after event onset) to allow for corticosteroids (if initiated) to be reduced to the equivalent of ≤ 10 mg/day oral prednisone. The acceptable length of the extended period of time must be agreed upon by the investigator and the Medical Monitor.

[b] If corticosteroids have been initiated, they must be tapered over ≥ 1 month to the equivalent of ≤ 10 mg/day oral prednisone before tiragolumab and atezolizumab can be resumed.

[c] Resumption of tiragolumab and atezolizumab may be considered in patients who are deriving benefit and have fully recovered from the immune-mediated event. Patients can be re-challenged with tiragolumab and atezolizumab only after approval has been documented by both the investigator (or an appropriate delegate) and the Medical Monitor.

Management Guidelines for Immune-Mediated Neuropathy

Event	Management
**Immune-mediated neuropathy, Grade 1**	Continue tiragolumab and atezolizumab.<br>Investigate etiology.
**Immune-mediated neuropathy, Grade 2**	• Withhold tiragolumab and atezolizumab for up to 12 weeks after event onset. [a]<br>Investigate etiology.<br>• Initiate treatment as per institutional guidelines.<br>• If event resolves to Grade 1 or better, resume tiragolumab and atezolizumab. [b]<br>• If event does not resolve to Grade 1 or better while withholding tiragolumab and atezolizumab, permanently discontinue tiragolumab and atezolizumab and contact Medical Monitor. [c]
**Immune-mediated neuropathy, Grade 3 or 4**	• Permanently discontinue tiragolumab and atezolizumab and contact Medical Monitor. [c]<br>• Initiate treatment as per institutional guidelines.
**Myasthenia gravis and Guillain-Barré syndrome (any grade)**	• Permanently discontinue tiragolumab and atezolizumab and contact Medical Monitor. [c]<br>• Refer patient to neurologist.<br>• Initiate treatment as per institutional guidelines.<br>• Consider initiation of corticosteroids equivalent to 1-2 mg/kg/day oral or IV prednisone.

[a] Tiragolumab and atezolizumab may be withheld for a longer period of time (i.e., > 12 weeks after event onset) to allow for corticosteroids (if initiated) to be reduced to the equivalent of ≤ 10 mg/day oral prednisone. The acceptable length of the extended period of time must be agreed upon by the investigator and the Medical Monitor. [b] If corticosteroids have been initiated, they must be tapered over ≥ 1 month to the equivalent of ≤ 10 mg/day oral prednisone before tiragolumab and atezolizumab can be resumed. [c] Resumption of tiragolumab and atezolizumab may be considered in patients who are deriving benefit and have fully recovered from the immune-mediated event. Patients can be re-challenged with tiragolumab and atezolizumab only after approval has been documented by both the investigator (or an appropriate delegate) and the Medical Monitor.

Management Guidelines for Immune-Mediated Meningoencephalitis

Event	Management
Immune-mediated meningoencephalitis, all grades	Permanently discontinue tiragolumab and atezolizumab and contact Medical Monitor. [a]<br>Refer patient to neurologist.<br>Initiate treatment with corticosteroids equivalent to 1-2 mg/kg/day IV methylprednisolone and convert to 1-2 mg/kg/day oral prednisone or equivalent upon improvement.<br>If event does not improve within 48 hours after initiating corticosteroids, consider adding an immunosuppressive agent.<br>If event resolves to Grade 1 or better, taper corticosteroids over ≥ 1 month.

[a] Resumption of tiragolumab and atezolizumab may be considered in patients who are deriving benefit and have fully recovered from the immune-mediated event. Patients can be re-challenged with tiragolumab and atezolizumab only after approval has been documented by both the investigator (or an appropriate delegate) and the Coordinator of the study.

Management Guidelines for Renal Events

Event	Management
**Renal event, Grade 1**	• Continue tiragolumab and atezolizumab.<br>• Monitor kidney function, including creatinine, closely until values resolve to within normal limits or to baseline values.
**Renal event, Grade 2**	• Withhold tiragolumab and atezolizumab for up to 12 weeks after event onset. [a]<br>Refer patient to renal specialist.<br>• Initiate treatment with corticosteroids equivalent to 1-2 mg/kg/day oral prednisone.<br>• If event resolves to Grade 1 or better, resume tiragolumab and atezolizumab. [b]<br>• If event does not resolve to Grade 1 or better while withholding tiragolumab and atezolizumab, permanently discontinue tiragolumab and atezolizumab and contact Medical Monitor. [c]
**Renal event, Grade 3 or 4**	• Permanently discontinue tiragolumab and atezolizumab and contact Medical Monitor.<br>Refer patient to renal specialist and consider renal biopsy.<br>• Initiate treatment with corticosteroids equivalent to 1-2 mg/kg/day oral prednisone.<br>• If event does not improve within 48 hours after initiating corticosteroids, consider adding an immunosuppressive agent.<br>• If event resolves to Grade 1 or better, taper corticosteroids over ≥ 1 month.

---

[a] Tiragolumab and atezolizumab may be withheld for a longer period of time (i.e., > 12 weeks after event onset) to allow for corticosteroids (if initiated) to be reduced to the equivalent of ≤ 10 mg/day oral prednisone. The acceptable length of the extended period of time must be agreed upon by the investigator and the Medical Monitor.

[b] If corticosteroids have been initiated, they must be tapered over ≥ 1 month to the equivalent of ≤ 10 mg/day oral prednisone before tiragolumab and atezolizumab can be resumed. [c] Resumption of tiragolumab and atezolizumab may be considered in patients who are deriving benefit and have fully recovered from the immune-mediated event. Patients can be re-challenged with tiragolumab and atezolizumab only after approval has been documented by both the investigator (or an appropriate delegate) and the Coordinator of the study.

Management Guidelines for Immune-Mediated Myositis

Event	Management
**Immunemediated myositis, Grade 1**	• Continue tiragolumab and atezolizumab.<br>• Refer patient to rheumatologist or neurologist.<br>• Initiate treatment as per institutional guidelines.
**Immunemediated myositis, Grade 2**	• Withhold tiragolumab and atezolizumab for up to 12 weeks after event onset [a] and contact Medical Monitor.<br>• Refer patient to rheumatologist or neurologist.<br>• Initiate treatment as per institutional guidelines.<br>• Consider treatment with corticosteroids equivalent to 1-2 mg/kg/day IV methylprednisolone and convert to 1-2 mg/kg/day oral prednisone or equivalent upon improvement.<br>• If corticosteroids are initiated and event does not improve within 48 hours after initiating corticosteroids, consider adding an immunosuppressive agent.<br>• If event resolves to Grade 1 or better, resume tiragolumab and atezolizumab. [b]<br>• If event does not resolve to Grade 1 or better while withholding tiragolumab and atezolizumab, permanently discontinue tiragolumab and atezolizumab and contact Medical Monitor. [c]
**Immunemediated myositis, Grade 3**	• Withhold tiragolumab and atezolizumab for up to 12 weeks after event onset [a] and contact Medical Monitor.<br>• Refer patient to rheumatologist or neurologist.<br>• Initiate treatment as per institutional guidelines. Respiratory support may be required in more severe cases.<br>• Initiate treatment with corticosteroids equivalent to 1-2 mg/kg/day IV methylprednisolone, or higher-dose bolus if patient is severely compromised (e.g., cardiac or respiratory symptoms, dysphagia, or weakness that severely limits mobility); convert to 1-2 mg/kg/day oral prednisone or equivalent upon improvement.<br>• If event does not improve within 48 hours after initiating corticosteroids, consider adding an immunosuppressive agent.<br>• If event resolves to Grade 1 or better, resume tiragolumab and atezolizumab. [b]
	• If event does not resolve to Grade 1 or better while withholding tiragolumab and atezolizumab, permanently discontinue tiragolumab and atezolizumab and contact Medical Monitor. [c]<br>• For recurrent events, treat as a Grade 4 event.
**Immunemediated myositis, Grade 4**	• Permanently discontinue tiragolumab and atezolizumab and contact Medical Monitor.<br>• [c] Refer patient to rheumatologist or neurologist.<br>• Initiate treatment as per institutional guidelines. Respiratory support may be required in more severe cases.<br>• Initiate treatment with corticosteroids equivalent to 1-2 mg/kg/day IV methylprednisolone, or higher-dose bolus if patient is severely compromised (e.g., cardiac or respiratory symptoms, dysphagia, or weakness that severely limits mobility); convert to 1-2 mg/kg/day oral prednisone or equivalent upon improvement.<br>• If event does not improve within 48 hours after initiating corticosteroids, consider adding an immunosuppressive agent.<br>• If event resolves to Grade 1 or better, taper corticosteroids over ≥ 1 month.

[a] Tiragolumab and atezolizumab may be withheld for a longer period of time (i.e., > 12 weeks after event onset) to allow for corticosteroids (if initiated) to be reduced to the equivalent of ≤ 10

mg/day oral prednisone. The acceptable length of the extended period of time must be agreed upon by the investigator and the Medical Monitor.

[b] If corticosteroids have been initiated, they must be tapered over ≥ 1 month to the equivalent of ≤ 10 mg/day oral prednisone before tiragolumab and atezolizumab can be resumed. [c] Resumption of tiragolumab and atezolizumab may be considered in patients who are deriving benefit and have fully recovered from the immune-mediated event. Patients can be re-challenged with tiragolumab and atezolizumab only after approval has been documented by both the investigator (or an appropriate delegate) and the Coordinator of the study.

Management Guidelines for Suspected Hemophagocytic Lymphohistiocytosis or Macrophage Activation Syndrome

**Event**	**Management**
**Suspected HLH or MAS**	Permanently discontinue tiragolumab and atezolizumab and contact Medical Monitor.<br>Consider patient referral to hematologist.<br>Initiate supportive care, including intensive care monitoring if indicated per institutional guidelines.<br>Consider initiation of IV corticosteroids, an immunosuppressive agent, and/or anticytokine therapy.<br>If event does not respond to treatment within 24 hours, contact Medical Monitor and initiate treatment as appropriate according to published guidelines (La Rosée 2015; Schram and Berliner 2015; La Rosée et al. 2019).<br>If event resolves to Grade 1 or better, taper corticosteroids over ≥ 1 month.

HLH = hemophagocytic lymphohistiocytosis; MAS = macrophage activation syndrome.
